# Validation of the Spanish Version of *Fear of COVID-19 Scale*: its Association with Acute Stress and Coping

**DOI:** 10.1007/s11469-021-00615-x

**Published:** 2021-10-08

**Authors:** J. A. Piqueras, M. Gomez-Gomez, J. C. Marzo, P. Gomez-Mir, R. Falco, B. Valenzuela, PSICORECUR-SOS COVID-19 study group, Raquel Falcó, Alfonso Lopez-Nuñez, Agustín E. Martínez-González, Juan Carlos Marzo, Ornela Mateu, Beatriz Moreno-Amador

**Affiliations:** 1 Department of Health Psychology, Faculty of Social and Health Sciences, Miguel Hernandez University, Avda. de la Universidad, s/n. Edf. Altamira, 03202 Elche, Alicante Spain; 2 Center for Applied Psychology, Miguel Hernandez University, Avda. de la Universidad, s/n. Edf. Altamira, 03202 Elche, Alicante Spain; 3 Centro Psytel-Psicología y Sexología and Centro Arela Logopedia, Madrid, Spain; 4 Centro Psicología y Salu and Universidad Nacional de Educación a Distancia, Madrid, Spain; 5 Grupo Profesional Psicológicamente, Praxis Psicología Integral, and Centro Integral de Desarrollo Infantil (CIDI Children), Santo Domingo, Dominican Republic

**Keywords:** COVID-19, Fear of COVID-19 scale, Stress, Coping, Psychometrics

## Abstract

The COVID-19 is a “unique” stressor, which can produce physical and psychological trauma. Coping styles can buffer this psychological impact. Consequently, this paper aims to psychometrically adapt the Fear of COVID-19 scale (FCV-19S) to Spanish and examines the relationships between FCV-19S, stress response, and coping strategies. The sample comprised a convenience sample of 1146 participants (12–83 years), 880 from Spain (76.8%), and 266 from Dominican Republic (23.2%). Overall, the findings support a one-factor structure for FCV-19S, consisting of 7-items, and was invariant across age, sex, occupational status, and cross-national. Therefore, indicating evidences of construct validity. Evidences of reliability were also observed (Cronbach’s *α* = .86, McDonald’s *ω* = .86, Guttmann’s *λ*6 = .86, greatest lower bound = .91, composite reliability = .85, and average variance extracted = .44). Moreover, as regards criterion-related validity, the mediation analysis indicated that the relationship between FCV-19S and acute stress was positive and high, with maladaptive coping styles mediating the relationship, and with a stronger mediation for men. The findings give evidences of the reliability and validity of the Spanish version of FCV-19S among Spanish-speaker participants, which provides the chance of cross-cultural studies.

At the time (05/30/2020), the 5,796,257 confirmed cases of COVID-19 and 362,483 deaths represent the reality of 216 countries around the world. The World Health Organization (WHO, 2020) reports that Spain has around 238,278 cases and 29,037 COVID-19-related deaths. Also, the Dominican Republic has 485 deaths out of 16,068 cases (WHO, 2020).

In a short period, COVID-19 has become a global pandemic that generates a huge economical (Cao et al., 2020) and psychological impact on the population (Mamun & Griffiths, 2020; Pakpour & Griffiths, 2020; Schimmenti et al., 2020; Wang et al., 2020a, b). Many people may experience fear, worry, and stress that affect their quality of life, as a consequence of the uncertainty about when an effective treatment or vaccine will be available (Harper et al., 2020; Satici et al., 2020). Also, some people may use maladaptive strategies such as alcohol and drug use (Lee, 2020).

Since its appearance in the city of Wuhan in December 2019, studies have been carried out to analyze the effect of this situation on the population. The decisions taken to prevent the spread of that disease, such as social isolation and/or quarantine (Chew et al., 2020) have been related to other psychosocial risks such as discrimination and stigmatization due to fear of contagion, according to what happened in previous pandemics such as SARS, Ebola, and H1N1 (Brooks et al., 2018; Lin, 2020), with emotional problems such as anxiety and depression with different levels of severity depending on the age (Huang & Zhao, 2020; Liang et al., 2020) and sex (Wang et al., 2020a, b; Zhang & Ma, 2020), and in different groups such as health professionals (Huang & Zhao, 2020). Furthermore, quarantine is the most predictive factor for symptoms of acute stress disorder and post-traumatic stress disorder (Brooks et al., 2020).

Considering that COVID-19 can be viewed as a unique stressor, according to the Lazarus and Folkman (1984) model of coping and stress, a high perceived impact and a low coping efficacy against the disease, tends to be associated with disarrangement in the perceived physical and psychological health (Cheng et al., 2006). According to Connor-Smith and Compas (2004), active coping responses directed with the stressor, or the thoughts and emotions associated with it, provoke a better adaptation. In contrast, avoidance or denial responses, which involve distancing oneself from the stressor and the thoughts, and emotions associated with it, were found to be associated with a worse adaptation process.

Recent studies focused on the impact caused by COVID-19 demonstrate the need to evaluate the fear response to this disease (Wang et al., 2020a, b), in order to develop effective interventions to cope with the situation and improve psychological recovery capacity (Wang et al., 2020a, b). Likewise, the possibility of measuring Fear of COVID-19 will be useful to analyze the consequences at a global level on populations’ mental health and the possibility of finding differences between a variety of countries.

Ahorsu et al. (2020) has developed a specific measure of Fear of COVID-19, *the Fear of COVID-19 scale* (FCV-19S) (Ahorsu et al., 2020). This is a 7-item questionnaire to assess the Fear of COVID-19. The FCV-19S scale (Ahorsu et al., 2020) has shown good psychometric properties such as internal consistency (*α* = 0.82) and acceptable test–retest reliability (ICC = 0.77). According to the Rasch analysis, its item separation reliability (0.99), item separation index (11.45), person separation reliability (0.77), and person separation index (2.82) were all satisfactory, indicating the test provides useful information about Fear of COVID-19. Six subsequent studies have supported their psychometric properties (Alyami et al., 2020; Bitan et al, 2020; Reznik et al., 2020; Sakib et al., 2020; Satici et al., 2020; Soraci et al., 2020).

The original study comprised 717 Iranian participants (> 18 years old). The authors reported invariance based on sex and age, as well as a significant relationship with depression (*r* = 0.42) and anxiety (*r* = 0.51) and with perceived infectibility and germ aversion of COVID-19 (*r* = 0.483 and *r* = 0.46, respectively).

The validation of this scale in other languages, such as Persian, Arabic, Hebrew, Russian, Bangla, Turkish, Greek, and Italian, presents a unifactorial structure (Alyami et al., 2020; Bitan et al., 2020; Reznik et al., 2020; Sakib et al., 2020; Satici et al., 2020; Soraci et al., 2020; Tsipropoulou et al., 2020), as well as factor invariance based on sex and age (Sakib et al., 2020); along with adequate internal consistency (*α* between 0.81 and 0.88), respectively (Alyami et al., 2020; Bitan et al., 2020; Reznik et al., 2020; Sakib et al., 2020; Satici et al., 2020; Soraci et al., 2020; Tsipropoulou et al., 2020). Only the study made by Bitan et al. (2020) supports a two-factor model of the scale, although the authors themselves indicate the one-factor model as a more parsimonious solution.

In addition, the FCV-19S has also received support in validation studies for evidence of validity. Thus, the different studies report a moderate size association of the FCV-19S with anxiety (*r* = 0.43), depression (*r* = 0.24), and stress (*r* = 0.33) of the abbreviated depression, anxiety and stress scales (DASS-21) (Bitan et al., 2020); the large size with anxiety (*r* = 0.66) and hospital anxiety and depression scale (HADS) (Alyami et al., 2020) depression (*r* = 0.56) and total score (*r* = 0.66) (Alyami et al., 2020); large magnitude with anxious-depressive symptoms of HADS (*r* = 0.65) and with a measure called “Severity Measure for Specific Phobia – Adult” (*r* = 0.70) (Soraci et al., 2020); of medium magnitude with depression evaluated with the patient health questionnaire-9 (PHQ-9) (*r* = 0.41) (Sakib et al., 2020); of medium to large magnitude with depression (*r* = 0.38), anxiety (*r* = 0.55) and stress (*r* = 0.47) of the abbreviated DASS-21 (Satici et al., 2020) and of medium magnitude with behavior change (*r* = 0.31), PROMIS anxiety (*r* = 0.20), reported risk (*r* = 0.31); care/harm (*r* = 0.20); purity/sanctity (*r* = . 25); quality of life physical (*r* = 0.37); and quality of life environmental (*r* = 0.31). The last published study has also showed large correlation with anxiety by generalized anxiety disorder-7 (GAD-7) (*r* = 0.71) and depression by PHQ-9 (*r* = 0.47) (Tsipropoulou et al., 2020).

Predictive models have also been carried out, where the Fear of Covid-19 predicts depression, anxiety, and depersonalization, as well as indirectly satisfaction with life through the mediation of anxiety and stress (Satici et al., 2020).

Some studies also indicate differences according to sex (higher scores in women) (Bitan et al., 2020; Reznik et al., 2020; Sakib et al., 2020; Tsipropoulou et al., 2020) and higher scores in university students vs. graduates (Reznik et al., 2020), while a single study indicates higher scores in people with low socioeconomic status, with chronic diseases, belonging to risk groups and with family members affected by COVID-19 (Bitan et al., 2020). Another study has showed that old people (over the age of 75), and participants with lower education displayed elevated Fear of COVID-19 (Tsipropoulou et al., 2020). Moreover, the study made by Reznik et al. (2020) indicates higher scores in Russian versus Belarusian participants.

The present study aims to assess the psychometric properties, reliability, and validity of the FCV-19S scale for the Spanish-speaking population. Specifically, we aimed to examine reliability estimates and evidences of construct validity (measurement model; measurement invariance and latent mean differences across age, sex, occupational status, and cross-national) and criterion-related validity by means of structural models to provide evidence of convergent-divergent validity describing the relations of FCV-19S with psychological impact (stress) and stress-coping strategies).

## Method

### Participants and Procedure

The current cross-sectional study was part of the international cross-cultural study “Psychological Impact of Confinement by COVID-19 in Spain & Dominican Republic.” The data were collected through online surveys. We reached 1405 participants, of which 1392 consented to participate voluntarily. Finally, 1146 cases were included in our study, as they had completed all the measures of variables under study and met the previously established inclusion criteria: (i) resident in Spain or Dominican Republic; (ii) aged 12 years or older; and (iii) being able to understand written Spanish. The mean age of the participants (*N* = 1146) was 35.39 years (SD ± 14.10). The 75.20% of the sample were females (*n* = 970), and around half of the participants were active employees (*n* = 636; 55.50%).

Specifically, participants were recruited from online advertisements, e-mail campaigns, blogs, social media, and SMS campaigns which covered the entire country. All procedures conducted were approved by the Ethics Committee of Miguel Hernández University (reference: DPS.JPR.01.20). Informed consent was obtained electronically before data were collected from the participants. Detailed information about the final sample for this study is presented in Table 1. Student subgroup included any kind of students, including university and vocational training students, as well as candidates for public office; active worker group consisted of full-time, part-time, employees, etc.; and inactive workers were unemployed, retired, housewife, temporary lay-offs of staff, etc.

**Table 1:** Participants’ characteristics (*N* = 1146)

Variables	*n* (%)		
	Spain	Dominican Republic	Total
Country of residence	880 (76.80)	266 (23.2)	880
Gender			
Male	236 (26.8)	48 (18.0)	284 (24.8)
Female	644 (73.2)	218 (82.0)	862 (75.2)
Age groups			
12–19	58 (6.6)	17 (6.4)	75 (6.5)
20–29	299 (34.0)	173 (65.0)	472 (41.2)
30–39	149 (16.9)	30 (11.3)	179 (15.6)
40–49	164 (18.6)	22 (8.3)	186 (16.2)
50–59	143 (16.3)	19 (7.1)	162 (14.1)
60–83	67 (7.6)	5 (1.9)	72 (6.3)
Occupational status			
Student	209 (23.8)	70 (26.3)	279 (24.3)
Active worker	479 (54.4)	157 (59.0)	636 (55.5)
Inactive worker	192 (21.8)	39 (14.7)	231 (20.2)

### Measures

In this study, measures of Fear of COVID-19, psychological impact (stress), and coping strategies were administered.

#### Fear of Covid-19

Spanish version of Fear of Covid-19 scale.

The Spanish FCV-19S assesses Fear of COVID-19 and was adapted from the English version of the scale published by Ahorsu et al. (2020). As recommended in the Standards (AERA, APA, and NCME, 2014), an iterative process involving translation and English–Spanish back translation was used. The screening tool consists of seven items (e.g., “I cannot sleep because I am worried about getting Covid-19”) with a 5-item Likert point response from 1 (strongly disagree) to 5 (strongly agree). The total score ranges from 7 to 35, with higher scores indicating a higher level of Fear of COVID-19. The psychometric properties of the Spanish version of FCV-19S are presented in the “Results” section.

#### Psychological Impact: Acute Post-traumatic Stress Disorder

##### Impact of event scale-revised

The psychological impact of COVID-19 was measured using the IES-R (Weiss, 1996). The IES-R is a 22-item questionnaire with 4-point Likert-type scale (0 = not at all, 1 = rarely, 3 = sometimes, 4 = often) composed of three subscales: avoidance, intrusion, and hyperarousal. The IES assesses subjective distress resulting from a traumatic life event. The Spanish version by Baguena et al. (2001) was administered, which has shown good psychometric properties. Alpha coefficient was 0.92 for the total score in this study.

#### Coping Strategies

##### The Brief Coping Orientation to Problems Experience

COPE-28 is an inventory of 28 items assessing how people handle stressful situations (COPE-28; Carver, 1997; Spanish adaptation of Morán et al., 2010). It measures 14 different stress-coping strategies using 28 questions (two questions for each strategy), which were clustered into adaptive or maladaptive strategies, as previously studies have defined (Carver, 1997; Connor-Smith & Compas, 2004; Kasi et al., 2012): adaptive stress-coping was formed by religion; active coping; planning; acceptance; positive reframing; instrumental support; emotional support; and humor; and maladaptive stress-coping included behavioral disengagement; denial; self-distraction; self-blame; substance use; and venting. Each question is answered using a 4-point Likert-type scale, ranging from 0 (I never do) to 3 (always do this). The maximum score of adaptive stress-coping is 64 points (16 questions covering eight strategies) and the maximum score of maladaptive stress-coping is 48 points (12 questions covering six strategies). Alpha coefficients were 0.68 for maladaptive stress-coping and 0.80 for adaptive stress-coping strategies in this study.

Slight adaptations were performed from the versions of IES-R and COPE-28 (changing verbal tenses where needed) to account for the nature of the stressful event explored and coping in response to COVID-19.

### Analysis

In order to validate the FCV-19S, analysis of psychometric properties included item analysis, reliability estimates (corrected item-total correlation, Cronbach’s *α*, McDonald’s *ω*, Guttmann’s *λ*6, composite reliability, and average variance extracted), and evidences of validity (construct validity: measurement model, measurement invariance across, latent mean differences; and criterion-related validity, specifically convergent-divergent validity by means of a structural model).

Structural equation model (SEM) was used to test the measurement model, measurement invariance, and latent mean differences across age, sex, country of residence, and occupational status (Byrne, 2006). SEM also was used to test structural model to describe the relations between characteristics of interest and provide evidences of convergent-divergent validity (variables: FCV-19S and measures of psychological impact (stress) and stress-coping strategies). All analyses, CFA and SEM, were carried out using the method of robust maximum likelihood (robust ML). We reported the following indices: chi-square (*χ*2), Satorra Bentler Chi-square (S-B *χ*2), robust root mean square error approximation (R-RMSEA), robust comparative fit index (R-CFI), and standardized root mean square residual (SRMR). For RMSEA, values less than 0.06 indicate a good fit model (Schumacker & Lomax, 2004). The R-CFI value indicate good fit with values greater or equal to 0.95 (Bentler, 1990), while the SRMR values are good with lower values to 0.08, and it is considered acceptable when values approach 0.10.

Factorial invariance of the model (FI) was analyzed following the procedure suggested by Byrne (2006), according to which the measurement invariance applies to (a) validity of the configural model (M0, base line model, no constrains), (b) metric invariance (equal factor loadings across groups, M1), (c) scalar invariance (equal item intercepts across groups, M2), and (d) item uniqueness invariance (equal item error variances/covariances across groups, M3). When the strong measurement invariance (metric and scalar) is reached, the comparison of latent means is justified. According to the methodology proposed by Cheung and Rensvold (2002), we reported R-CFI, ∆CFI, R-RMSEA, and SRMR. A value of ∆CFI smaller than or equal to 0.01 indicates that the null hypothesis of invariance should not be rejected. After these considerations, the calculations to compare the latent means across sex were carried out.

The analyses were carried out using the following statistical packages: IBM SPSS 25.0 (IBM Corp., Armonk, NY), EQS 3.0 and JASP 0.11.1.

## Results

### Descriptive Data

**Fig. 1:**
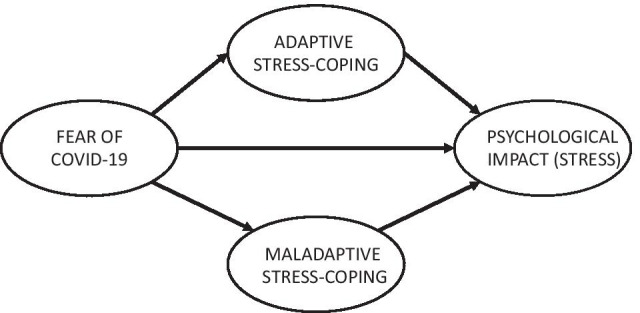
Theoretical path models between Fear of COVID-19, stress-coping strategies, and psychological impact (stress)

Item analysis results for FCV-19S are given in Table 2. Most items had skewness and kurtosis values within the ± 2.0 range, but items 3 and 6 showed kurtosis higher than 4, not confirming that they were normally distributed (Table 2 and Fig. 1). Mean score of FCV-19S was 15.17 (SD = 5.88).

**Table 2:** Descriptive statistics and item-total correlation of the Fear of COVID-19 scale (*N* = 1146)

Item	Corrected item-total correlation	*M* (SD)	Skewness	Kurtosis
Item 1. I am most afraid of COVID-19	0.64	3.13 (1.26)	− 0.18	− 9.99
Item 2. It makes me uncomfortable to think about COVID-19	0.65	2.76 (1.29)	0.16	− 1.09
Item 3. I worry a lot about COVID-19	0.54	1.35 (0.72)	2.40	6.04
Item 4. I am afraid of losing my life because of COVID-19	0.63	2.16 (1.32)	0.87	− 0.47
Item 5. When I am watching news and reading stories about COVID-19 on social media, I become nervous or anxious	0.64	2.59 (1.31)	0.32	− 1.03
Item 6. I cannot sleep because I’m worrying about getting COVID-19	0.62	1.44 (0.85)	2.09	4.11
Item 7. My heart races or palpitates when I think about getting COVID-19	0.70	1.74 (1.12)	1.43	0.99
Average score of items		2.17 (1.12)

## Evidences of Validity

### Measurement Model

CFA analysis showed adequate fit values for the entire sample and across sex, age groups, country of residence, and occupational status (see Table 3). However, certain indices, such as the R-RMSEA were somewhat higher than 0.06, specifically for the 30–39-year-old group and the student’s group. This values only indicate an acceptable fit model (Schumacker & Lomax, 2004), but the remaining values were adequate. The R-CFI value indicate good fit with values greater or equal to 0.95 (Bentler, 1990), while the SRMR values are good, with lower values to 0.08.

**Table 3:** Goodness-of-fit indices in the confirmatory factor analyses Fears COVID

Model	^2^	SB^2^	*df*	R-CFI	R-RMSEA (90% CI)	SRMR	*ω*	*α*	*λ*6	AVE	CR
Total	61.38	48.80	10	0.982	0.058 (0.042–0.093)	0.022	0.864	0.863	0.862	0.442	0.845
Men	26.67	18.35	10	0.980	0.054 (0.003–0.093)	0.032	0.855	0.855	0.860	0.416	0.829
Women	46.82	38.89	10	0.983	0.058 (0.039–0.078)	0.022	0.863	0.862	0.860	0.442	0.845
12–19 y/o	21.65	18.28	10	0.951	0.064 (0.000–0.136)	0.056	0.847	0.844	0.842	0.443	0.846
20–29 y/o	29.40	24.49	10	0.981	0.055 (0.028–0.084)	0.028	0.855	0.853	0.851	0.424	0.834
30–39 y/o	25.95	25.12	10	0.957	0.092 (0.047–0.138)	0.032	0.878	0.877	0.876	0.685	0.902
40–49 y/o	18.44	16.39	9	0.986	0.067 (0.000–0.117)	0.034	0.881	0.880	0.896	0.682	0.901
50–59 y/o	20.28	13.02	11	0.994	0.034 (0.000–0.093)	0.038	0.854	0.853	0.858	0.650	0.892
> 60 y/o	10.63	12.17	10	0.987	0.055 (0.000–0.145)	0.035	0.877	0.876	0.897	0.673	0.899
Spanish	46.23	36.50	10	0.984	0.055 (0.036–0.075)	0.022	0.863	0.862	0.862	0.673	0.902
Dominican R	16.79	13.97	10	0.993	0.039 (0.000-0.082)	0.025	0.870	0.863	0.866	0.672	0.905
Students	27.01	24.35	10	0.967	0.072 (0.036–0.108)	0.035	0.849	0.848	0.846	0.673	0.897
Workers	44.32	32.85	10	0.983	0.060 (0.038–0.083)	0.027	0.873	0.872	0.874	0.673	0.906
Non-workers	22.61	20.29	10	0.975	0.067 (0.022-0.109	0.031	0.873	0.872	0.874	0.673	0.898

SB^2^, Satorra–Bentler scaled chi-square; *R-CFI*, robust comparative fit index; *R-RMSEA*, robust root mean square error of approximation; *CI*, confidence interval; *SRMR*, standardized root mean square residual; *ω*, McDonald's; *α*, Cronbach’s *α*; *λ6*, Gutmann’s; *AVE*, average variance extracted; *CR*, composite reliability

Fit indices were all within the acceptable limit and factor weights between 0.47 and 0.86 for the total sample and across groups (see Table 4).

**Table 4:** Factor loadings in the confirmatory factor analysis for latent variables of the Fears COVID-19

Item	Total	Men	Women	12–19 y/o	20–29 y/o	30–39 y/o	40–49 y/o	50–59 y/o	> 60 y/o	Sp^a^	RD^b^	STD^c^	Wor^d^	Nwor^e^
1	0.54	0.48	0.55	0.69	0.58	0.60	0.47	0.50	0.66	0.52	0.60	0.59	0.52	0.54
2	0.58	0.56	0.57	0.64	0.59	0.56	0.57	0.56	0.67	0.57	0.59	0.60	0.57	0.55
3	0.63	0.67	0.62	0.53	0.57	0.73	0.72	0.63	0.55	0.64	0.62	0.54	0.66	0.62
4	0.66	0.67	0.66	0.65	0.61	0.76	0.68	0.68	0.82	0.67	0.63	0.63	0.68	0.64
5	0.62	0.56	0.63	0.79	0.62	0.55	0.60	0.63	0.76	0.59	0.71	0.67	0.62	0.57
6	0.75	0.73	0.75	0.71	0.68	0.77	0.81	0.79	0.61	0.76	0.73	0.64	0.80	0.73
7	0.83	0.79	0.83	0.62	86	0.83	0.93	0.76	0.64	0.84	0.81	0.81	0.84	0.84

^a^Spanish sample; ^b^Dominican Republic sample; ^c^Students; ^d^workers; ^e^non-workers

The analyses indicated that the measure was invariant according to age, sex and country of residence (see Table 5), so that we can assert that measurement invariance was reached for all comparisons.

**Table 5:** Invariance constraints for the Fears COVID-19

Model	*χ*^2^	SB*χ*^2^	*df*	R-CFI	ΔR-CFI	R-RMSEA (90% CI)	SRMR
M0 gender	73.49	44.80	18	.987		.051 (.032–.070)	.027
M1 gender	79.47	58.06	26	.984	−.003	.046 (.030–.062)	.040
M2 gender	122.10	89.80	32	.985	.001	.056 (.043–.070)	.048
M3 gender	124.98	91.91	36	.986	.001	.052 (.039–.065)	.047
M0 age	136.79	102.07	54	.981		.068 (.048–.088)	.040
M1 age	200.55	153.89	83	.971	−.010	.067 (.050–.083)	.096
M2 age	349.85	318.45	123	.962	−.009	.091 (.079–.104)	.099
M3 age	386.81	349.72	143	.958	−.004	.087 (.075–.099)	.107
M0 residence	63.03	41.82	18	.990		.048 (.029–.067)	.024
M1 residence	68.02	47.50	24	.990	.000	.041 (.024–.059)	.034
M2 residence	115.07	86.29	32	.989	−.010	.054 (.041–.068)	.035
M3 residence	123.12	99.98	36	.986	−.003	.044 (.029–.059)	.038
M0 occupation	93.94	66.97	27	.983		.056 (.038–.074)	.031
M1 occupation	139.56	97.33	39	.974	.009	.059 (.043–.074)	.069
M2 occupation	193.15	159.39	55	.969	−.005	.068 (.053–.083)	.071
M3 occupation	199.95	165.05	63	.970	.001	.061 (.043-.075)	.074

*SBχ*^*2*^, Satorra–Bentler scaled chi-square; *R-CFI*, robust comparative fit index; *R-RMSEA*, robust root mean square error of approximation; *CI*, confidence interval; *SRMR*, standardized root mean square residual; *ΔR-CFI*, R-CFI difference; *M0*, free model (baseline); *M1*, M0 with invariant factor loadings; *M2*, M1 with invariant intercepts; *M3*, M2 with invariant factor variances and covariances

Since the strong measurement invariance (metric and scalar) was reached, the comparison of latent means across sex, age, country of residence, and occupational status was justified. As can be seen in Table 6, results showed higher means on Fear of COVID-19 for females, for Dominican participants and for workers versus students. Regarding differences between age groups, the extreme groups had the lowest scores of Fear of COVID-19, which is adolescents and the elderly. Therefore, lower scores were found for adolescents compared to the rest of age groups, except for older than 60. The 20–29 group had lower scores than the 40–49 one, but higher than the older than 60 s. The 40–49 group also showed higher scores than the older than 60 s global score of stress residence groups on Fear of COVID-19.

**Table 6:** Latent mean differences across sex, age, country of residence, and occupational stutus

	Fears COVID				
*Men* (Reference)	0.00				
*Women*					
Mean estimate (ME)	0.237				
Standard error (SE)	0.048				
Test statistic (TS)	4.90^*^				
*Spain* (Reference)	0.00				
*Dominican Republic*					
Mean estimate (ME)	0.127				
Standard error (SE)	0.055				
Test statistic (TS)	2.30^*^				
*Students* (Reference)	0.00				
*Workers*		(Reference)			
Mean estimate (ME)	0.125				
Standard error (SE)	0.048				
Test statistic (TS)	2.60^*^				
*Non-workers*					
Mean estimate (ME)	0.047	− 0.070			
Standard error (SE)	0.072	0.052			
Test statistic (TS)	0.658	− 1.362			
*12–19-year-olds* (Reference)	0.00				
*20–29-year-olds*		(Reference)			
ME	0.180				
SE	0.084				
TS	2.14^*^				
*30–39-year-olds*			(Reference)		
ME	0.285	0.090			
SE	0.089	0.074			
TS	3.22^*^	1.22			
*40–49-year-olds*				(Reference)	
ME	0.330	0.156	0.081		
SE	0.073	0.069	0.075		
TS	4.52^*^	2.31^*^	1.08		
*50–59-year-olds*					(Reference)
ME	0.279	0.051	− 0.025	− 0.102	
SE	0.087	0.068	0.079	0.067	
TS	3.19^*^	0.750	− 0.318	− 1.56	
≥ *60-year-olds*					
ME	0.141	− 0.181	− 0.204	− 0.251	− 0.150
SE	0.119	0.076	0.092	0.073	0.089
TS	1.19	− 2.37^*^	− 2.33^*^	− 3.46*	− 1.72

## Path Models

To further examine the associations among Fear of COVID-19 and psychological impact (stress) as well as the role of a protective variable (stress-coping strategies), a structural model was conducted (see Table 7, and Fig. 1). Before performing the path analysis, we analyzed the correlation matrix of the FCV-19S with the IES-R and COPE-28. The correlation matrix showed that the FCV-19S score was associated with psychological impact measured as global score of stress by IES-R (0.59), as well as with maladaptive coping strategies (0.25) of COPE-28. There was not association with adaptive coping strategies (− 0.03).

**Table 7:** Goodness-of-fit indices in the predictive model

Model	*χ*^2^	SB*χ*^2^	*df*	R-CFI	R-RMSEA (90% CI)	SRMR
Total (I)^a^	2833.03	2542.48	247	0.721	0.090 (0.087 − 0.093)	0.102
Total (II)^b^	682.73	589.82	97	0.905	0.067 (0.061 − 0.072)	0.057
Men (I)^a^	909.30	803.33	247	0.713	0.089 (0.082 − 0.096)	0.114
Men (II)^b^	248.46	210.41	96	0.918	0.075 (0.063 − 0.086)	0.072
Women (I)^a^	2131.25	1928.14	247	0.722	0.089(0.085 − 093)	0.077
Women (II)^b^	520.45	456.96	97	0.907	0.066(0.060–0.072)	0.101

^a^Includes Fear of COVID-19, adaptive and maladaptive stress-coping strategies and Stress; ^b^includes Fear of COVID-19, maladaptive stress-coping strategies and stress. *SBχ*^*2*^, Satorra–Bentler scaled chi-square; *R-CFI*, robust comparative fit index; *R-RMSEA*, robust root mean square error of approximation; *CI*, confidence interval; *SRMR*, standardized root mean square residual

As expected, the analysis revealed a good fit to the data when we entered Fear of COVID-19, psychological impact, and only maladaptive stress strategies, not when both adaptive and maladaptive strategies were included (see models 1 in Table 7).

**Fig. 2:**
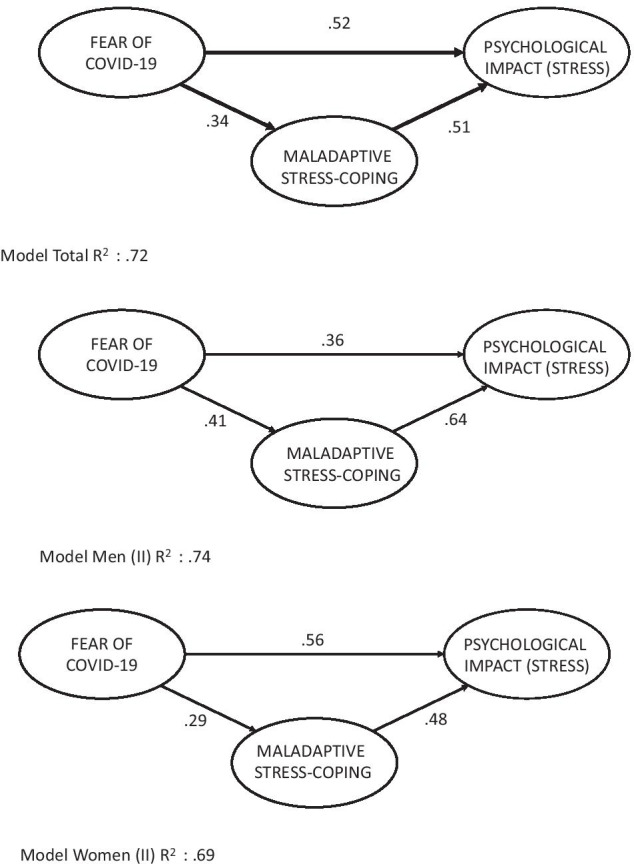
Path models between Fear of COVID-19, stress-coping strategies, and psychological impact (stress) for total, women and men groups. (all paths *p* < 0.01)

The theoretical model can be seen in Fig. 1. The contrasted models for the entire sample, and for both men and women can be seen in Fig. 2. For the total sample, the relationship between Fear of COVID-19 and psychological impact was significant (percentage of variance explained = 72%), with mediating effect of maladaptive coping strategies. For men, the relationship was even stronger (74%), being the role of maladaptive stress-coping higher than direct effect, while for women there was a stronger direct effect between Fear of COVID-19 and impact, along with an indirect effect through maladaptive coping strategies. The model for women explained 63% of the total explained variance.

## Reliability Estimates

After the confirmatory factor analysis, different types of reliability (i.e., internal consistency) were investigated to analyze the reliability of the measure and internal consistency. Therefore, all items (see Table 2) had positive and acceptable corrected item-total correlation (0.54 to 0.70), within the range recommended between 0.20 and 0.70. by Streiner et al., (2015, p. 84). The internal consistency estimates of the FCV-19S in the entire sample was Cronbach’s *α* = 0.86, McDonald’s *ω* = 0.86, Guttmann’s *λ*6 = 0.86, Greatest lower bound = 0.91, composite reliability = 0.85, and average variance extracted = 0.44. Estimates values for each subgroup were high and equivalent to the entire sample, in all cases with internal consistency estimates higher than 0.84 (see Table 3).

## Discussion

The COVID-19 pandemic is undoubtedly the greatest current health and economic problem for humanity (Cao et al., 2020; Lei et al., 2020; Peng et al., 2012).

There is no doubt that the disease has an impact on physical health, but a growing number of studies indicate that the impact on mental health is also being felt (Huang & Zhao, 2020; Lai et al., 2020; Lei et al., 2020). For these reasons, having assessment tools to investigate the effects of COVID-19 on people’s mental health is relevant.

In this study, the results indicated an average score of items of 2.17 ± 1.12, lower than that reported by Ahorsu et al. (2020), reporting a mean score of 3.81 ± 1.04, as well as a mean total score on the Spanish FCV-19S (15.17 ± 5.88) slightly lower than those reported by previous studies: 16.86 ± 6.06 by Soraci et al. (2020); 22.75 ± 5.65 and 20.29 ± 5.90 for female and male, respectively, by Sakib et al. (2020); and 17.2 ± 4.7 by Reznik et al. (2020) or 14.69 ± 4.98 and 17.43 ± 5.06 for male and female, respectively, by Tsipropoulou et al. (2020).

In relation with factorial structure, this measure has a unidimensional structure, consistent with all previous studies in different languages (Ahorsu et al., 2020; Bitan et al., 2020; Reznik et al., 2020; Sakib et al., 2020; Satici et al., 2020; Soraci et al., 2020; Tsipropoulou et al., 2020).

As regards factorial invariance, our study confirms invariance by sex and age, consistent with Ahorsu et al. (2020) or Sakib et al. (2020), and extend knowledge on the invariance across country of residence (Spain and Dominican Republic), and occupational status (students, active population, and inactive population).

Finally, validity evidences indicate that Fear of COVID-19 is a predictor of acute post-traumatic stress disorder, although there is mediation by maladaptive coping strategies that explain part of the effect of the relationship. This finding supports the concurrent validity of the instrument and is consistent with other data on predictive models, in which the Fear of COVID-19 predicts depression, anxiety, and depersonalization, as well as indirectly life satisfaction, through the mediation of anxiety and stress (Satici et al., 2020). The correlation between FCV-19S and stress and maladaptive stress-coping strategies, with an effect size large and medium, respectively, were also coherent with previous data that show FCV19S total score is associated with anxiety, depression, stress, and behavior change and quality of life-related variables (Alyami et al., 2020; Bitan et al., 2020; Sakib et al., 2020; Satici et al., 2020; Soraci et al., 2020; Tsipropoulou et al., 2020), also with effect size between medium and large.

Similarly to previous studies (Bitan et al., 2020; Reznik et al., 2020; Sakib et al., 2020; Tsipropoulou et al., 2020), we found that the Fear of COVID-19 was higher for females. However, we did not find higher scores in undergraduates vs. graduates as reported by Reznik et al. (2020), but we did find higher scores in middle age adulthood versus adolescents, emerging adulthood and elderly as well as in active workers versus students. Our data did not support the finding by Tsipropoulou et al. (2020), who reported higher scores on Fear of COVID-19 for the elderly and people with lower education. Participants from Dominican Republic also showed higher scores than Spaniards. Reznik et al. (2020) also found differences between Russian versus Belarusian participants. Anyway, in all cases, the differences were small.

In relation to reliability estimates, our study provides adequate internal consistency supported by different coefficients (between 0.84 and 0.91), which is consistent with previous studies reporting values between 0.81 and 0.88 (Ahorsu et al., 2020; Alyami et al., 2020; Bitan et al., 2020; Sakib et al., 2020; Reznik et al., 2020; Satici et al., 2020; Soraci et al., 2020). Additionally, our study provides reliability data for different sexes, age groups, occupational status, and places of residence.

It is worth mentioning that this study is not exempt from some limitations, such as having followed a convenience sampling, carrying out the analyses with a sample where women are more represented, where the population of Spain is also more represented and where not all the variables that may be affecting the Fear of COVID-19 have been controlled. Even so, the sample is large, the origin is varied, and it can be said that it has allowed the test to be validated.

In summary, this study has at least 4 new contributions: to make available to the Spanish-speaking community, the second worldwide in extension, a measure that allows the assessment of Fear of COVID-19; to provide evidence of validity and estimates of reliability that support the psychometric properties of the measure in people between 12 and 83 years, extending the range of applicability of the measure, since for the first time it has been applied to people under 18 years; it supports the unidimensional structure of the measure; and it provides support for the relationship between Fear of COVID-19 and acute post-traumatic stress disorder, taking into account a mediating variable such as coping strategies.
